# Spanish media coverage of youth mental health issues during the COVID-19 pandemic

**DOI:** 10.1186/s12888-023-05054-7

**Published:** 2023-08-10

**Authors:** Juan Pablo Carrasco, Anne-Marie Saucier, Rob Whitley

**Affiliations:** 1 Deparment of Psychiatry, Hospital Clínico Universitario de Valencia, 46010 Valencia, Spain; 2 Instituto de Investigación Sanitaria del Hospital Clínico de Valencia (INCLIVA), Valencia, Spain; 3 Douglas Mental Health University Institute, Verdun, QC Canada; 4 Department of Psychiatry, McGill University, Montreal, QC Canada

**Keywords:** Stigma, Mass media, Mental health, Mental disorder, Youth, Child

## Abstract

**Background:**

The media portrayal of mental health is relevant in shaping the population’s attitudes towards mental disorders. However, there is little information about the representation of young mental health issues in the Spanish-language press, especially in the context of the COVID-19 pandemic. The general objective of this study was to analyse the tone and content of Spanish newspaper articles about mental disorders in youth during the COVID-19 pandemic.

**Methods:**

We collected media articles from the 10 most read news sources over a 6 month period (January-June 2021). These articles were coded for content using a standardised codebook, followed by an inductive thematic analysis. A total of 205 news items were evaluated.

**Results:**

Results showed that the majority of the news items had an overall positive tone (68.3%), only 5.4% were stigmatising and only 7.3% were related to violence. However, few articles offered help seeking information (6%), adolescents were rarely quoted (14%) and children were never quoted. Substantial differences are described in terms of age, gender and disorder. The thematic analysis led to three emergent themes: (i) violence and victimisation; (ii) the COVID-19 pandemic; and (iii) technology and social media.

**Conclusions:**

The percentage of news in the Spanish media that refer to young people’s mental health in a stigmatising way or in a way associated with violence is very low. Furthermore, the COVID-19 pandemic may have promoted more positive discussion about youth mental health. However, major challenges remain to be addressed, as patients are seldom quoted, very few articles offer help-seeking information, and a narrative of victimisation without appropriate discussion of resilience regularly occurs.

## Background

### Mental health stigma in the media

Stigma towards mental disorders continues to be a serious problem in the field of mental health. People who suffer from mental health problems have to deal not only with the inherent difficulties of the disorder itself but also with discrimination and rejection in several different ways [1]. Defining stigma as a social and structural process of power imbalance [2], people with mental health problems are almost continuously suffering the consequences of status loss associated with mental illness. In this process, stereotypes in which people with mental health problems are labelled as “different”, “inferior”, or “violent” [3] are maintained through different factors. One of the main factors is the portrayal of mental disorders in the media. Frequently, sensational, violence-related and one-dimensional portrayals of mental disorders, and people with mental health problems, create and perpetuate inaccurate perceptions and erroneous stereotypes that contribute to stigma, fear, and prejudice [4, 5]. However, evidence suggests that the representation of mental disorders in the media vary considerably depending on different realities. One of the most studied is the variation according to the language or location of the media. There is an extensive bibliography analysing this phenomenon in the different English-speaking countries, while a smaller but growing number of studies have been carried out in the Spanish-speaking countries [6]. Interesting work done in Chile [7], Spain [8, 9] and in other Spanish-speaking countries [10], suggest that Spanish-language press may be less stigmatising than the English-language press. However, greater bibliographic support is necessary to be able to affirm clear differences in communication media based on language.

### Particularities of stigma in young people

Another factor that is regularly studied in research on media and mental health is variations in coverage according to the characteristics of the protagonists of the news story. Among these, age, gender and type of mental disorder are some of the most significant. In this regard, there is little literature in this field that examines how young people with mental disorders are portrayed in the media [7, 11]. The existing literature converges to indicate that: (i) youth mental health coverage tends to include more elements of responsible journalism than adult mental health coverage, often in devoted feature articles rather than episodic news coverage [12, 13]; (ii) young people are a more sensitive and impressionable group and are therefore at greater risk of exposure to harmful information [14]; (iii) youth are often portrayed as victims of violence [15]; and (iv) there is little emphasis on prevention, intervention or resources [16]. This literature, however, has certain limitations. Some articles are quite dated [11, 12], and all are focused on the Anglophone media. To our knowledge, there has been no specific analysis of media coverage of youth mental health issues in Spain or Spanish-speaking countries.

At the same time, evidence suggests that portrayals of adolescents and youth may vary according to important demographic variables. For example, some research suggests that adolescents are more often associated with violence than children [17], however this remains under-researched. Similarly, several studies have shown that media coverage of adult men with mental health issues tends to be more stigmatising than media coverage of adult women with mental illness [18], but there has been little research examining gender differences in media portrayals of youth with mental health issues. In terms of clinical characteristics, existing research implies that schizophrenia, bipolar disorder and post-traumatic stress disorder tend to be described in a more stigmatising manner (e.g. linked to crime and violence), while anxiety and depression are more likely to be described in positive and recovery-oriented terms [19, 20]. But again, these comparisons have been made in media that discuss adult mental health. To our knowledge, none of these comparisons have been made in children and adolescents with mental illness.

The above situation becomes particularly relevant when examining the emerging literature on the relationship between digital communication and mental health in youth. For example, recent research indicates a contagion effect of negative emotions or suicidal behaviour amongst young people using social media [21, 22]. Conversely, there are also studies describing the possible protective effect of supportive and supervisory interventions on social media [23, 24] against suicidal behaviour. Within all the discussion surrounding the use of social media by young people, one conclusion emerges with a broad consensus. This is that the content and manner of communications and interactions can influence mental health outcomes, both on social media [25] and in the traditional media [26].

### The covid-19 pandemic and mental health communication

In addition to all the above, another factor that is fundamental when analysing representations of mental disorders in the media is the socio-cultural epoch. It is therefore of particular interest what impact the covid-19 pandemic, which dominated community life for several years, may have had in this area. As reflected in early studies, the COVID-19 pandemic has had a huge impact on the field [27]. This has been described until now in different aspects. On the one hand, communication about the state of the pandemic has influenced the mental health status of the population [28], with health communication becoming one of the most discussed topics in the media. On the other hand, mental health communication itself has changed in relation to the pandemic. The putative increase in mental health problems has also been reflected in an increase in mental health communication in the media [29]. However, the literature on whether this increase has led to more or less responsible communication about mental health is contradictory [30, 31]. This increase in mental health problems has been particularly acute in the young population [32, 33]. It is hypothesised that, among other factors, this may have been due to the great impact of periods of isolation and changes in academic rhythm at a time when socialisation is essential [34]. Nevertheless, no work has specifically analysed mental health media coverage focused on children or adolescents during the pandemic. Which, as discussed above, would be of great interest for further study.

Given the above-described situation, the primary aim of this study is to examine the tone and content of media articles about youth mental health in Spain during the COVID-19 era. A secondary aim is to examine differences in portrayal according to demographic and diagnostic variables such as sex, age and mental health disorder. This will fill a gap in the literature and can be used as a springboard for new interventions aimed at improving media coverage of these issues.

## Methods

### Design

A cross-sectional descriptive study of the news related to child (0 to 13 years old) and adolescent (14 to 18 years old) mental health that have appeared in the Spanish print and online news media was conducted. Standard procedures for the analysis of news content in the media [35] were followed. It involved systematically collecting news articles, and subsequently two analyses (quantitative deductive and qualitative inductive ones) were conducted.

Firstly, in the deductive analysis, a standardised codebook adapted from the previous work of other authors [7, 9, 36, 37] (described in detail in Table 1) was created based on the most similar previous literature on content analysis of mental health stories in the media [36, 37], using information from previous articles on Spanish-language press [7, 9]. In addition, it was contextualised with the recommendations of the Canadian guidelines on child and adolescent mental health communication [38]. Secondly, an inductive qualitative thematic analysis of the articles was also performed. In this, a content analysis was carried out based on previous studies, after which a code of labels and themes was generated, as will be detailed below.

**Table 1:** Codebook

*General descriptive information*		
Title of the newspaper		Literal name
Date of story		Eight-digit format (e.g. 01012000)
Retrieval type		1. Newspaper 2. Online web news
Length		Number of words
Scope		(1) National (2) Regional
Placement of piece		0. Online (1) Front page (2) Inner page
Disorder or “disease”		Referred in the story
What the news is due to?		1. The situation of a person with a mental disorder who affected himself/herself 2. The situation of a person with a mental disorder that affected others 3. Dissemination article about the disorder or mental health 4. Article about a specific scientific breakthrough/event in relation to the disorder or mental health
Who is the news focused on?		(1) Children (until 13 included) (2) Adolescents (from 13 to 18) 3. Both 4. Other
Who are the protagonists of the news?		0. Unspecified/None (1) Women/Girls (2) Men/Boys 3. Both
Is the sexual orientation or gender identity of the protagonists mentioned in the story?		0. Unspecified (1) Yes, homosexual (2) Yes, bisexual 3. Yes, transexual
*Key Questions*		
Is the general tone on mental health optimistic/positive?	(1) Optimistic/positive (2) Negative/pessimistic	
Is recovery or resilience/hope a significant theme?	0. No 1. Yes	
Does the story stigmatize in tone and/or content?	0. No 1. Yes	
Is danger, violence or crime negatively linked to mental disorder?	0. No 1. Yes	
Are mental health experts or mental health community leaders directly/indirectly cited in the text (paraphrased)?	0. Not quoted 1. Positively quoted 2. Negatively quoted 3. Mix quoted (positively-negatively)	
Are children or adolescents with mental health problems directly/indirectly quoted in the text (paraphrased)?	0. Not quoted 1. Positively quoted 2. Negatively quoted 3. Mix quoted	
Are the relationships (family, friends, etc.) of the person with a mental health problem directly/indirectly quoted in the text (paraphrased)?	0. Not quoted 1. Positively quoted 2. Negatively quoted 3. Mix quoted	
Are mental health interventions discussed in the story?	0. Not discussed 1. Positively discussed 2. Negatively discussed 3. Positively and negatively discussed	
Is there a biological or psychosocial approach to the aetiology and/or treatment of the mental health problem?	0. Not discussed 1. Biological approach 2. Psychosocial approach 3. Biological and psychological approach	
If suicide is mentioned, which explanation does the article give?	0. Not mentioned 1. Personal 2. Familiar 3. Biological 4. Environmental 5. Multicausal 6. No explanation	
Is the scarcity of resources or the poor quality of care for mental health an issue?	0. No 1. Yes	
Does the article provide help-seeking information?	0. No 1. Yes	
Does the article try to educate the public about suicide or mental health?	0. No 1. Yes	
Does the article draw over-simplified links about the influence of social media, video games, social, financial or gender issues-seek facts?	0. No 1. Yes	

### Search strategy and news selection

The five most read print newspapers [39] and the five most read online News websites [40] were selected. El País, El Mundo, La Vanguardia, Abc and La Voz de Galicia were the chosen print newspapers; ElEspañol.com, Eldiario.es, La razón.es, El nacional.cat and Diario de Sevilla were the chosen online sources.

**Fig. 1:**
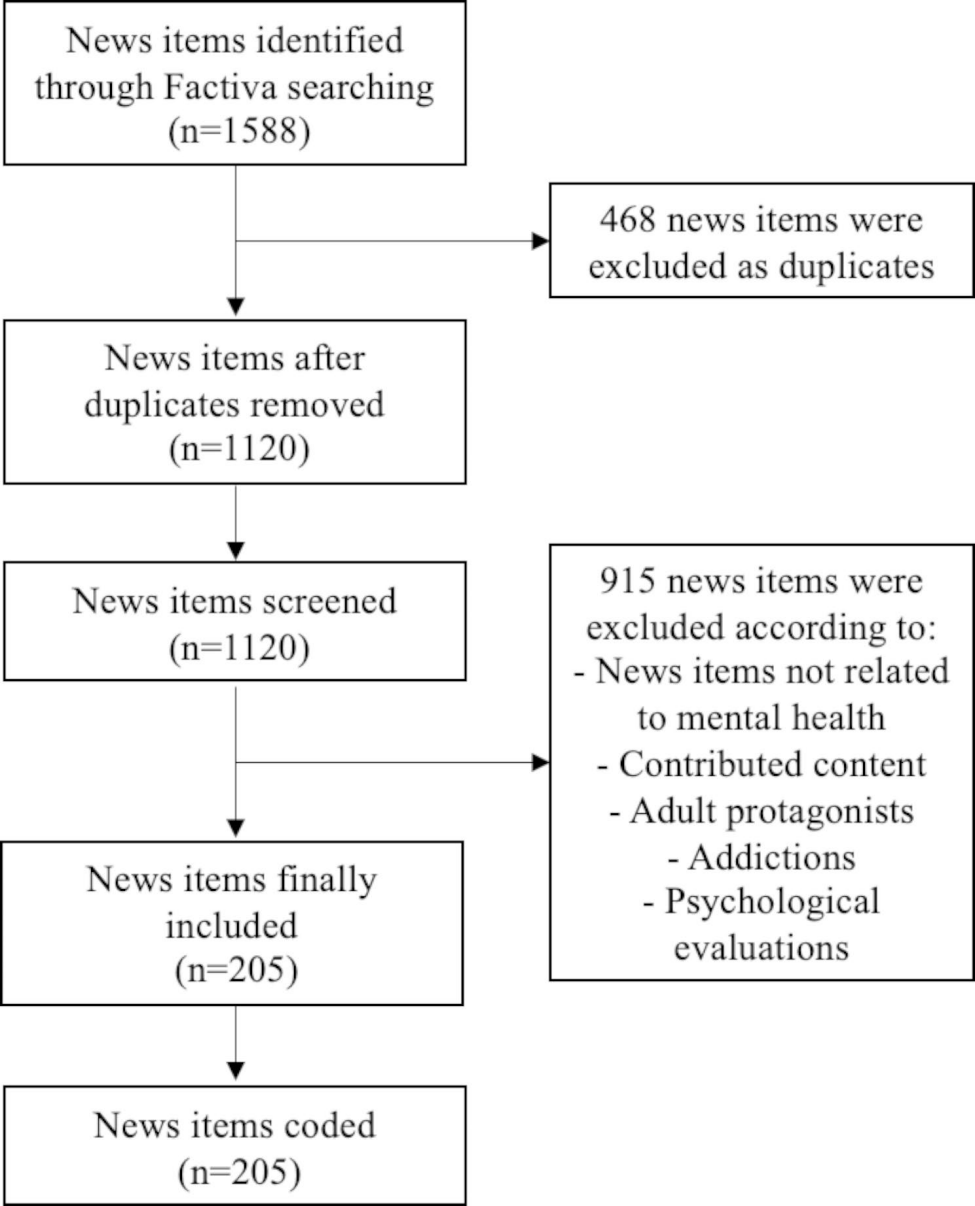
Selection flowchart of news items

The search engine Factiva was used to identify and retrieve the news. The time range defined for the search was January 1 2021 to June 30 2021, a representative period during the COVID-19 pandemic. This period was chosen because it corresponds to the 3rd wave of the pandemic in Spain [41]. This wave had the second highest incidence of covid-19 cases and also one of the periods with the highest mortality from the disease [41]. Moreover, 12 months had already passed since the beginning of the pandemic, and its consequences on the mental health of the population had already begun to be described both in the scientific literature [42] and in the press [30, 31]. Six months was chosen as this provided a time window to examine patterns in an unexplored area, which has been used in previous studies [43, 44]. The news items that included the following keywords in the title, subtitle and/or body of the publication were selected: [suicid* or salud mental or psiquiatr* or trastorno* mental*] and [infan* or adolescen* or joven* or juven* or niñ*]. In English: [suicid* or mental health or psychiatr* or mental* disorder*] and [child* or adolescen* or youth* young* or boy* or girl*]. Contributed content (e.g. opinion pieces, letters to the editor, advice columns, etc.), news articles focusing mainly on adults and those using keywords in an irrelevant way were excluded. In addition, articles about drug addiction or alcohol related issues or psychiatric or psychological evaluations related to these issues were also excluded, as has been done in previous articles [7, 9, 36, 37]. This has been decided due to the particularities and differences in the description of such disorders in the press compared to others [45], which would have substantially broadened and blurred the objective of the analysis. As well, low prevalence among children and adolescents of these disorders have been described. Using the selected keywords, a total of 1,588 news items were retrieved. 468 were excluded for being duplicates articles, and 915 were excluded according to the other exclusion criteria already defined. The remaining 205 news items were successfully coded by the first author. The selection flowchart can be seen in Fig. 1.

### Deductive analysis

The first author received extensive training with the senior author of the article before coding. As part of the training, 20 media articles were consulted and coded in a pilot test to clarify coding procedures. This led to an inter-rater reliability test with the answers to 10 key questions. For this purpose, the Cohen’s kappa coefficient was used. For the first 10 pilot-coded news items, an average value of 0.54 was obtained between the first and second author, indicating a medium level of agreement. Further face-to-face training and discussion sessions were held with the last author to clarify concepts and solve discrepancies, after which a new set of 10 news items were coded and a value of 0.81 was obtained, indicating high level of agreement.

In the coding procedure, the first author read each news item and the last author read a sample (10%) of the articles, with arising issues discussed during regular supervision meetings, as an extra check and balance on the validity of coding. This methodology was chosen to enrich the skills of the first author, with 10% considered an adequate sample given the previous high inter-rater reliability test during the training stage. All codes were recorded in an Excel database created from the content of the codebook, which was exported to SPSS software version 25.0 [46] to facilitate analysis. Table 1 shows the codebook used. Once all articles were coded, frequencies and percentages were calculated for each variable, including comparisons of variation between articles focused on different demographics and different disorders.

### Inductive analysis

Moreover, an inductive qualitative thematic analysis of the articles was also performed. This followed standard procedure of identifying and labelling common themes that regularly appeared in the newspaper articles through systematic step-by-step thematic extraction [47–49]. More specifically, this involved labelling portions of the articles (or sometimes whole articles) with codes that summarised key content. All authors then discussed the coding and mapping efforts, paying particular attention to codes that reappeared regularly across the data set. At this stage, the authors consensually created a list of themes and sub-themes that best described the widespread patterns in the data [50]. The first author then engaged in a second round of supervised ‘focused coding’, utilising the thematic framework to formally enumerate the presence or absence of the identified themes and sub-themes in each article.

The themes and sub-themes presented in the results represent this framework and were agreed upon consensually by all the authors as a faithful summary of the common themes across the dataset. Data for this study was a collection of short media articles rather than lengthy in-depth interviews or focus groups transcripts. As such, the analysis was done manually rather than with computer-assisted qualitative data analysis software.

## Results

### Deductive analysis

A total of 205 news items were analysed. As can be seen in Table 2, most articles have an overall positive tone and are recovery oriented. As for public stigma, in the vast majority of news items no link was found between mental disorder and categories of violence, danger or criminality and only a few articles were stigmatising in tone or content. Nonetheless, the scarcity of resources, which is an aspect of structural stigma, is only discussed in 17.6% of news items. Furthermore, only 2.9% of news items provide help seeking-information.

**Table 2:** Distribution of frequencies and percentages for general categories on stigmatization, help-seeking, education purposes and protagonist quotes

	Yes		No	
	N	%	N	%
Is the general tone on mental health optimistic/positive?	140	68.3	65	31.7
Is recovery or resilience/hope a significant theme?	137	66.8	68	33.2
Does the story stigmatize in tone and/or content?	11	5.4	194	94.6
Is danger or violence negatively linked to mental disorder?	15	7.3	190	92.7
Is the scarcity of resources for mental health an issue?	36	17.6	169	82.4
Does the article provide help-seeking information?	6	2.9	199	97.1
Does the article try to educate the public?	112	54.6	93	45.5

Regarding quotes of experts, children or adolescents with a psychiatric diagnosis and their families, positive quotes greatly predominate compared to negative ones (negative quotes represent less than 4% in each category). In addition, quotes from experts are included much more frequently than those from patients or relatives, who are very rarely quoted (Table 3).

**Table 3:** Distribution of frequencies and percentages for general categories on protagonist quotes

	Yes, positively		Yes, negatively		No	
	N	%	N	%	N	%
Are mental health experts quoted in the text?	113	55.1	2	1	90	43.9
Are patients quoted in the text?	23	11.2	5	2.5	177	86.3
Are families quoted in the text?	16	7.8	10	4.9	179	87.3

**Fig. 2:**
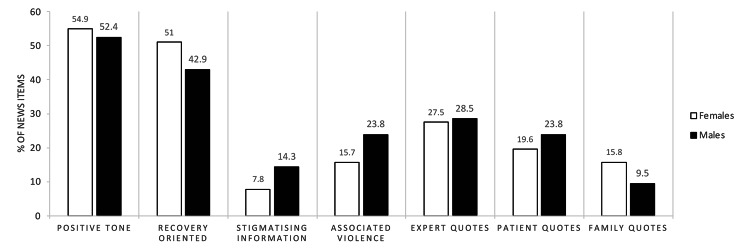
The figure shows the percentages (relative frequencies) of news items grouped by those whose protagonist is male or female, for the most relevant categories in Tables 2 and 3

**Fig. 3:**
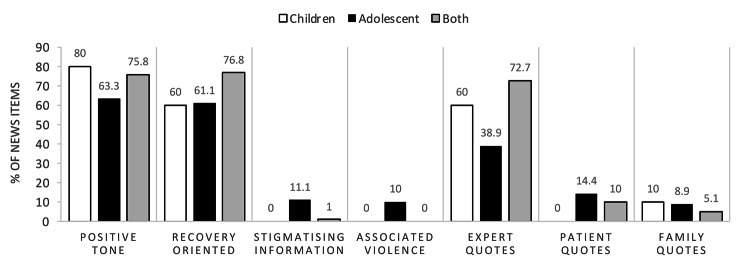
The figure shows the percentages (relative frequencies) of news items grouped by those whose protagonist is a child, an adolescent or both, for the most relevant categories in Tables 2 and 3

**Fig. 4:**
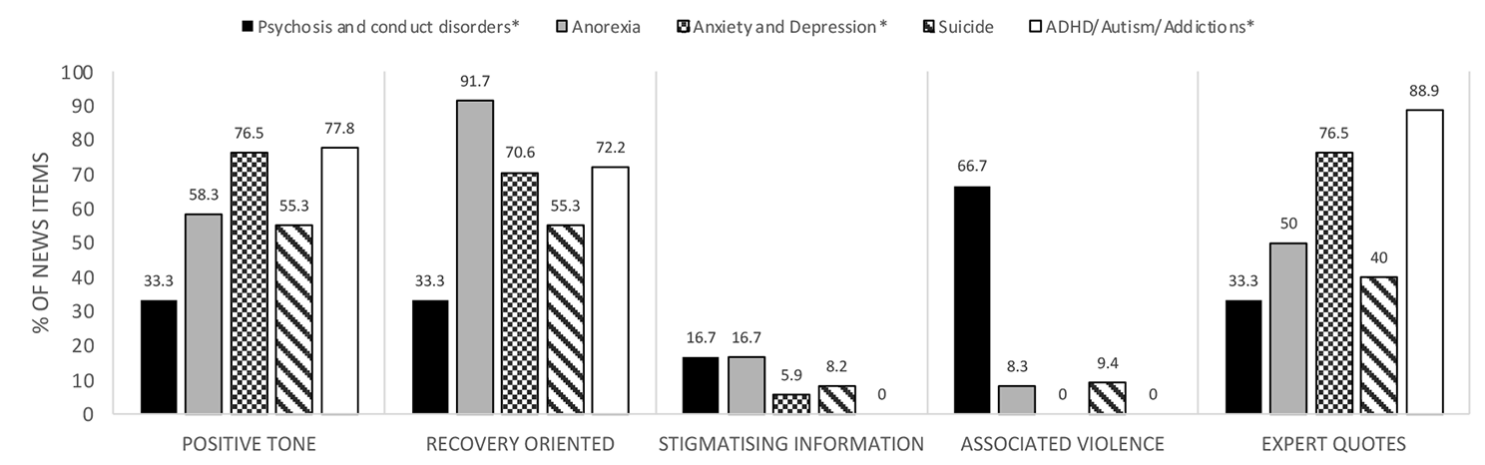
The figure shows the percentages (relative frequencies) of news items grouped by those whose protagonist is a young person diagnosed with psychosis or conduct disorders, anorexia, anxiety or depression, suicidal ideation or behaviour, and ADHD or autism or videogame addictions, for the most relevant categories in Tables 2 and 3

Consistent with the research question, the previously described results in Tables 2 and 3 were sub-analysed in a series of stratified analyses. First, we stratified by age in three groups, namely (1) news items whose protagonists are children; (2) news items whose protagonists are adolescents; and (3) news items dealing with both stages. Secondly, we stratified by gender in two groups, namely (1) news items whose protagonists are males; and (2) news items whose protagonists are females. Thirdly we stratified by mental disorder in five groups, namely news items whose protagonists are diagnosed from the following disorders: (1) psychosis and conduct disorders; (2) anorexia; (3) anxiety and depression; (4) suicide; and (5) neurodevelopmental disorders and videogame addictions. This is shown in Figs. 2, 3 and 4. In these analyses, we documented and compared frequencies and percentages of coding scores according to the above-described groupings. The description of the results according to the gender of the news protagonists can be seen in Fig. 2. Males are typically discussed in a more negative tone, less recovery oriented, more related to violence and in a more stigmatising way than females. In terms of quoting, males are quoted more often than females, but on the contrary, female relatives are more usually quoted than male relatives.

In relation to age, the majority of the articles are focused on adolescents and only a few are centred on children. In these, children are described in a more positive way compared to adolescents and adolescents are described as more violent and in a more stigmatising way, as shown in Fig. 3. In addition, children are never quoted and adolescents are only quoted in 10% of the articles.

Regarding the distribution of disorders covered, most news items discuss general issues in child or adolescent mental health (45.5%) or suicide (33.6%). When a specific disorder is mentioned, the most frequently discussed are anxiety and depression (6.7%), anorexia (4.7%) and internet gaming addiction (3.6%). Neurodevelopmental and gaming addiction disorders are described in a more positive tone and no news in these articles are related to violence nor stigma. Anorexia, anxiety and depression also tend to be portrayed in generally positive terms (see Fig. 4). Psychosis and conduct disorders were described most negatively, with the majority of articles about these disorders related to violence. In addition, patients and relatives are rarely quoted in discussions of any disorder.

### Inductive analysis

**Fig. 5:**
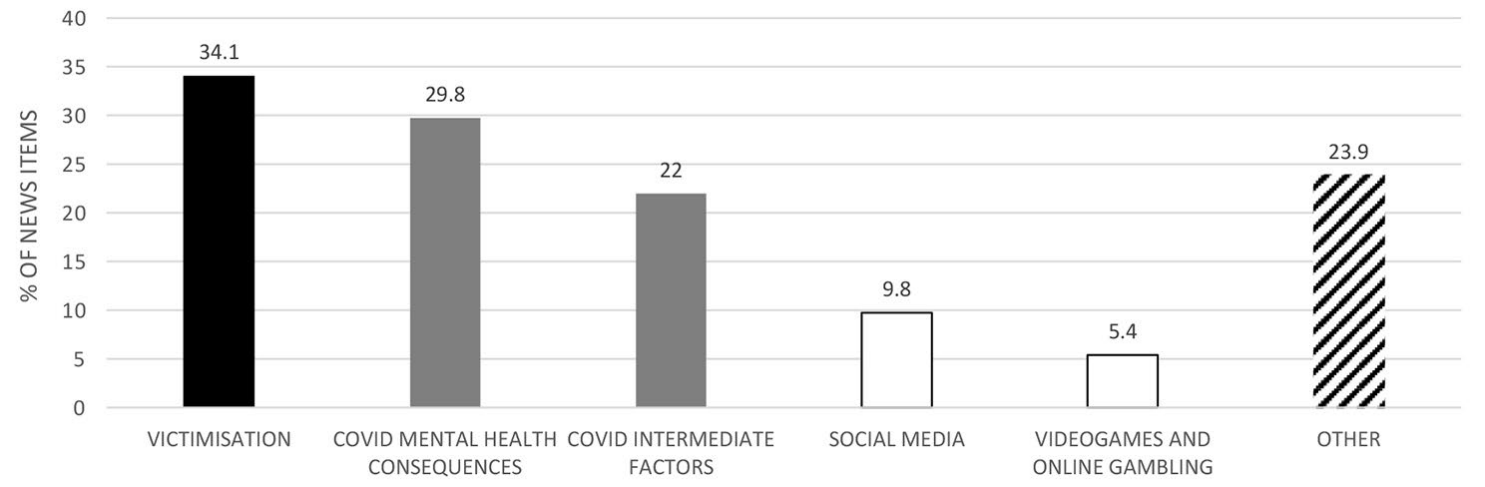
Percentage of articles in which the described sub-themes are discussed (out of the total number of articles, N = 205)

In terms of the thematic analysis, three main themes were observed: (i) violence and victimisation; (ii) the COVID-19 pandemic; and (iii) technology and social media. The frequency of the sub-themes can be seen in Fig. 5.

### Violence and victimization

A total of 70 articles (34.1%) contained information related to being a victim of violence and developing mental health problems. Most of these articles were about sexual abuse or family violence and, consequently, suffering different kinds of mental disorders (trauma, anxiety, depression, anorexia or personality/conduct disorders). For example, one article stated, “*My father was alcoholic and violent, my mother was very young, she couldn’t protect me and in the end I ended up hospitalised in psychiatry*” (El País, February 11th, 2021). These articles mainly recounted stories, with few educational information beyond the story and a lack of focus on resilience and help-seeking information.

Other news items focused on school bullying, as for example, “*A 15-year-old girl committed suicide on May 19 in Barcelona. The family states that she suffered bullying*” (La Razón, June 10th, 2021). These articles were usually recovery oriented and discussed more broader social issues: *“Bullying is not just a children thing, it can be the cause of anxiety or depression, which is why it is crucial that we all do our bit to fight it.”* (La Razón, April 28th, 2021). Articles about LGTBQI victimisation were also found. They were sharply divided into two narratives. On one hand talking about the mental health issues of transsexual people as a consequence of social discrimination, “*A teacher made me cry until the last day for treating me like a woman*” (El País, June 30th, 2021). On the other hand, focusing on “gender dysphoria” as a mental health disorder in itself.

Finally, news about institutional and police violence were observed, primarily concerning migration issues and how the institutional response carried out by the police could play a role in trauma, anxiety and depression among migrant children, such as: *“the mental health problems we most commonly observed among displaced children are related to symptoms of anxiety, depression and post-traumatic stress disorder due to the violence they have witnessed”* (elDiario.es, January 25th, 2021).

### COVID issues

The COVID-19 related articles were mainly focused on the mental health consequences of the pandemic. The majority of these articles delved into the intermediate factors and social consequences of the pandemic as they relate to mental health (66.2%, n = 68). In these, the social consequences of the pandemic, such as isolation, family changes, bereavement, loss of schooling, etc., are described as an intermediate factor between the pandemic and the increase in mental health problems in the child population. An example of this could be the following: *“Among the factors that explain this is the growing unease that the pandemic has created among young people, whether through isolation, death of family members or economic problems.”* (elDiario.es, May 6th, 2021). However, some news only described the mental health consequences without a broader discussion or while drawing simplified links, as for example, “*Anxiety over COVID-19 disrupts the lives of children and young people”* (La Voz de Galicia, January 31st, 2021). The main mental health consequence described was general emotional distress and when a specific disorder or mental health problem was mentioned, anxiety, anorexia and suicide were those most frequently linked to the pandemic.

Likewise, when intermediate factors and further explanations for mental health problems were discussed, the effect of lockdown and social restriction measures on child and adolescent socialisation was commonly described. For instance, *“Social contacts and participation activities have decreased, this greatly affects mood and emotional issues”* (elDiario.es, February 2nd, 2021).

Furthermore, economic problems arising from the closing of businesses, economic slowdown due to COVID-19 and school adaptation problems during and after lockdown were also related to mental health problems: *“45% of young people are sad and anxious because they cannot pay their rent, or their studies, which has worsened since the start of the pandemic”* (El Nacional, February 26th, 2021). It stands out that only 7.3% (n = 68) include clear information on resilience or recovery opportunities related to the pandemic, for example *“One of the keys to being more resilient in this crisis is to find new purposes that transcend the individual”* (El Mundo, April 7th, 2021).

### Technology and social media

Technology, particularly social media, was the most frequent element related to mental health. Most of these articles on technology and social media were related to suicide, self-harm and the “contagion” effect, as for example: “*If you are considering committing suicide, you will receive many messages in forums or on social networks about other people who, like you, also want to commit suicide”* (La Vanguardia, March 27th, 2021). Most articles with this theme were focused on the possible consequences of social media, but only a few of them included help-seeking information or advice on how to use them in a safer way.

Less frequently, video games and online gambling were also discussed as potential risk factors of mental health disorders, a few times describing them related to violence: *“He even skipped class so he could stay on the computer. He had mood swings, he changed his behaviour, he was violent”* (ABC, March 31st, 2021). Notably, only a few articles about technology (16.2% N = 31) highlighted possible benefits: *“technology plays a fundamental role in our society and is an aid to relationships and to learning”* (La Razón, February 7th, 2021).

## Discussion

To our knowledge, this is the first study of its kind examining Spanish-language media portrayals of youth mental health, and one of very few studies investigating media coverage of mental health per se in the Spanish-language media. The key finding of this study is that most Spanish media articles about youth mental health are positive, with the majority of stigmatising and violence-associated news stories focusing on adolescents, males, psychotic disorders and conduct disorders.

In general terms, compared to the previous generic research on media and mental health (without an age focus) published in Chile [7] or Spain [8, 9], the results were better for tone and recovery. In addition, the news items analysed were less associated with violence or crime compared to previous English-language data focused on children [12] but also less associated with recovery or broader social issues.

### Dissertation of results by age and gender

Despite these positive findings, there were some differences in portrayal according to age, gender and diagnosis of the protagonists. The results presented confirm that just as mentally healthy adolescents are more frequently associated with stereotypes of violence than mentally healthy children in the news media [17], this is also the case when the adolescent has a mental disorder. News items involving teenagers scored worse on all the variables in the study than news items involving children. This reflects a continuum in which adolescents with mental health problems are represented more similarly to adults with this kind of problems, i.e. in a more stigmatising and violent way, and young children with mental health problems in a more paternalistic way. Similarly, female youth with mental health problems were portrayed more positively in the media than males’ ones, which provides evidence in favour of the “chivalry hypothesis” posited by Whitley et al. [18], in this case applying to female children and female adolescents. In turn, females are quoted less than males as protagonists, but in the articles in which they do appear, more experts and family members are quoted.

### Dissertation of results by disorder

Regarding disorder described, the negative results of psychosis and conduct disorder converges with most of the previous literature without an age focus [7, 51, 52]. In this case, it confirms that these negative depictions also occur in young people diagnosed with psychotic or conduct disorders. The same is true for the generally more positive results of descriptions in news items of protagonists suffering from anxiety or depression [7, 20]. The results of our study are consistent with previous literature on this case also.

The results on mental disorders that are more typically associated with the young population are particularly interesting. ADHD in the present study is generally described in a positive way. These results are consistent with a similar US study that includes more years of evaluation of print media (from 1985 to 2008) [12], where people diagnosed with ADHD are described in a generally positive light. However, more recent studies published in Belgium, analysing the written media [53] and pictures on the internet [54] show more negative results in the representation of ADHD patients. Such results with negative stereotypes about ADHD are worrying, given the fact that a recent study has shown negative self-views and depressive symptomatology as a mediator of functional difficulties at academic level in this population [55]. In the case of autism, the positive results are also in line with previous literature [7, 56, 57]. However, some recent research indicates a lack of diversity in newspaper and television portrayals of people with autism [58, 59]. The results of the present study diverge from the existing literature most significantly in reports of anorexia and video game addiction. In the present study, the news items analysed generally describe these diagnoses in a neutral or positive way. However, in the previous literature, negative stereotypes and violence are more frequently described [60–62]. This could be due to the fact that in the aforementioned studies these diagnoses are analysed in information seen on the Internet, while in our study only the written press was reviewed.

### Dissertation of the inductive analysis results

With respect to the information described in the inductive analysis, the patterns of reporting regarding the COVID-19 in the present study were largely positive. This suggests the pandemic may have opened a window of opportunity for greater and more considered discussion of mental health issues. Many articles link the mental health problems due to the pandemic with broader issues, particularly interpersonal and social isolation, which have a crucial role in adolescent development [63, 64]. However, the results described in the section on technology and social networks are not as positive as those described above for the COVID-19 pandemic. The vast majority of news items talk about them only in a negative way. Moreover, there is no in-depth discussion of the responsible use of social media [21, 22], which would be of great interest. Neither are the positive points described, nor the opportunities they offer for communication and mutual support [65]. Given that the reality is that more and more children are using social networks to show their discomfort [66], it is of greater interest to focus efforts on how to take advantage of this reality for prevention and treatment interventions, rather than blanket criticism.

Although the findings are largely positive, there remains some room for improvement. The voices of adolescents are hardly ever included in the news items, and as for children, no article was found that included their opinion. These results are much worse regarding the inclusion of the protagonists than those of literature published by analysing Spanish or English-language press in other countries [7–9, 36]. Similarly, very few articles offer help-seeking information. Moreover, the portrayal of young people with mental health problems as victims is very frequent in the press analysed. All the above converges with a problem pointed out by different studies in the English-speaking countries [15–17]; that portrayal of youth with mental disorders as victims proceeds without enough emphasis on prevention, interventions or resources, thus creating a social image of children with mental health problems as helpless victims without capacity for agency. Likewise, the lack of inclusion of statements from children and adolescents and the much more frequent utilisation of quotes from experts and relatives makes the victimisation even more problematic, narrating the stories of youth in a paternalistic way. This could be due to, among other reasons, to the absence of anti-stigma campaigns targeting important stakeholder such as journalists in Spain. In the scientific literature, there is only one described campaign carried out in the early 2010s in Catalonia (one of Spain’s largest and richest regions) called “obertament” [67]. In this campaign, messages were sent through the Catalan media deconstructing stereotypes about mental health. However, this was a population intervention rather than a targeted intervention to stakeholders, meaning there was no specific section of the campaign targeting journalists. In the English-language press, there are several examples of campaigns focused on journalists in this sense in Canada [68], or the UK [69], for example, with very successful results. This difference between the English and Spanish-language press has been noted previously in the literature [10].

### Limitations

This research has some limitations to consider. First, only 6 months of news during the 3rd wave of the COVID-19 pandemic were analysed. This limited time span is a weakness in that a longer period may have led to additional findings. Furthermore, this period represents very special social circumstances due to the influence of the onset of the covid-19 pandemic. The extrapolation of these results to other contexts and periods is limited by the described situation. However, the number and diversity of newspapers used are a good representation of Spanish media and clear patterns with a high number of articles were found. Second, only online news and print news press were considered. Television, radio and social media networks such as Facebook and Instagram, which are a key source of people’s expression on diverse topics, were excluded from analysis. This may have skewed the results in a positive way, as although there is room for improvement, journalism professionals may have undergone some training in ethics (in general and even related to mental health issues), and also have to pass editorial gatekeepers when posting content. However, anyone can post almost any type of content on social media, (sometimes anonymously) meaning that negative stereotypes about young people’s mental health problems may be more prevalent in such outlets. In this sense, content analysis in social media and comparison with our results is a future line of research of great interest. Third, we did not conduct statistical tests comparing articles by age, gender and diagnosis. This is due to various factors, including low cell sizes and multiple (non-hypothesis driven) comparisons. This means that the results are only indicative and give a lay of the land for further formal testing in larger samples with purpose driven hypotheses.

### Implications

Different implications could be derived, both in the research and journalistic fields, from the present study. In the research field, to our knowledge the present study is the first to analyse child mental health in the Spanish-language press, and it would be of great interest for similar studies to be conducted in other Spanish-language countries in Latin America [70], where stigma remains a large issue. The study implies that more research is also necessary on media content related to ADHD, autism, anorexia and technology addiction, given that our findings diverge most from the previous literature in relation to these disorders commonly associated with young people. This could include prospective cross-national studies examining media content of these issues among different countries, for example a comparison of European nations or Spanish-speaking countries.

Moreover, further examining the association of technology with negative stereotypes, and the growing literature on the importance of technology in the lives and mental health of young people, is important, given the study findings. The analysis of information published on mental health in social media is itself an area of high potential. But probably of even greater concern is the interaction of users who talk about their problems in these networks and their moments of greatest vulnerability and risk. These approaches can provide great advances in understanding both the particularities of the contagion effect in the new forms of communication, and the opportunities for prevention and intervention through these media.

In the field of journalism, targeted interventions on stigma prevention and mental health education aimed at journalists would be of great value, given the results of the study. This could include the development and dissemination of recommendations and guidelines on reporting youth mental health issues such as including help-seeking information (e.g. help line numbers), trying to source and quote young people in stories about their mental health, and avoiding stigmatizing content such as linking mental illness to violence.

## Conclusions

The present study suggests that the percentage of news in the Spanish media that refer to young people’s mental health in a negative way or in a way associated with violence is very low. Furthermore, it seems that the COVID-19 pandemic has promoted more positive discussion about child and adolescent mental health and related interpersonal factors. However, major differences have been found in mental health portrayal by age, gender and mental disorder, with adolescents, males and patients with psychosis and conduct disorders more negatively represented. In addition, young people with mental health problems are often portrayed as victims, their voice is rarely included in the news, and there is not sufficient help-seeking information for news consumers. This suggests the need for brief educational interventions targeted at Spanish journalists to ensure the improvement of such important areas as those described above.

## Data Availability

The data that support the findings of this study are available from JPC but restrictions apply to the availability of these data, which were used under license for the current study, and so are not publicly available. Data are however available from the authors upon reasonable request and with permission of JPC.

## Declarations

### Authors’ information

JPC is a junior researcher based in Valencia, Spain, starting different research in social psychiatry, focusing on suicide, stigma and child psychiatry.

AS is a junior researcher based in Montreal, Canada, starting different research in social psychiatry, focusing on the representation of mental disorders in the media.

RW is a senior researcher based in Montreal, Canada, with extensive research experience in various fields of social and community psychiatry, including the portrayal of mental disorders in the media.
